# Successful Surgical Repair of Ventricular Septal Defect in a Child With Ocular Myasthenia Gravis and History of Myeloid Sarcoma Post‐HSCT: A Multidisciplinary Approach

**DOI:** 10.1002/ccr3.71589

**Published:** 2025-12-01

**Authors:** Shuhan Rong, Wanyu Xu, Xin Li

**Affiliations:** ^1^ Cardiothoracic Surgery Department Children's Hospital of Soochow University, Suzhou Jiangsu Province China

**Keywords:** hematopoietic stem cell transplantation, multidisciplinary management, ocular myasthenia gravis, pediatric cardiac surgery, ventricular septal defect

## Abstract

A 3 year‐old child with ocular myasthenia gravis and a history of hematopoietic stem cell transplantation underwent successful ventricular septal defect repair through carefully coordinated multidisciplinary management. The tailored anesthetic approach and infection‐control strategy enabled smooth recovery without neuromuscular or immunologic complications. This case demonstrates the feasibility of safe cardiac surgery in pediatric patients with rare, high‐risk comorbidities.

## Key Clinical Message

Successful cardiac surgery is feasible in children with ocular myasthenia gravis and post‐HSCT status through meticulous multidisciplinary management.

## 1 Introduction

Ventricular septal defect (VSD) represents the most prevalent congenital cardiac anomaly, and its surgical repair is a well‐established procedure with excellent outcomes [1]. However, the perioperative management becomes significantly more complex in pediatric patients presenting with rare comorbidities that independently pose substantial risks during major surgery. Myasthenia gravis (MG), particularly its ocular form (OMG) in young children, heightens susceptibility to respiratory complications and neuromuscular blocking agent (NMBA) sensitivity, potentially leading to prolonged mechanical ventilation or myasthenic crisis [2] Furthermore, a history of hematopoietic stem cell transplantation (HSCT) for hematologic malignancy, such as myeloid sarcoma, introduces challenges related to potential immunosuppression, increased infection susceptibility, and altered immune reconstitution, even in patients without active graft‐versus‐host disease (GVHD) or ongoing immunosuppression [3]. The confluence of these conditions—congenital heart disease requiring cardiopulmonary bypass, an autoimmune neuromuscular disorder affecting the acetylcholine receptor, and a post‐transplant immunologic state—creates a unique and high‐risk perioperative scenario. To the best of our knowledge, this report details the first successful surgical repair of a VSD in a child with coexisting OMG and a history of myeloid sarcoma successfully treated with HSCT, highlighting the critical importance of meticulous multidisciplinary planning.

## 2 Case History / Examination

In November 2022 (age 2 years), he presented with a 4 month history of a cervical mass. Bone marrow aspiration revealed hypoplastic granulopoiesis with MN1‐FLI1 fusion and mutations in CCND3, TP53, and CYYR1. Surgical biopsy confirmed myeloid sarcoma (CD33+, CD117+).

Between November 2022 and April 2023, he received sequential chemotherapy with MAE, HAM, and EA regimens. A transient pulmonary infection occurred during the treatment course but resolved after appropriate therapy.

On June 1, 2023, he underwent HLA 10/10–matched umbilical cord blood hematopoietic stem cell transplantation (HSCT) after conditioning with busulfan and cyclophosphamide (BU + CTX). GVHD prophylaxis included MMF and CSA. Neutrophil engraftment occurred on day +13, and immune reconstitution was completed by July 2023, with no GVHD or infection observed.

During a chemotherapy interval in February 2023, the patient developed ocular myasthenia gravis (OMG), presenting with bilateral ptosis and diurnal fluctuation. The neostigmine test was positive, while antibody and EMG studies were negative. He was treated with oral pyridostigmine (2.5 mg/kg per dose, three times daily), achieving stable symptom control.

By May 2025 (2 years post‐HSCT), preoperative multidisciplinary evaluation confirmed normal immune function, hematologic remission, and stable OMG (MGFA class I). Cardiac function was preserved, with a Ross score of 1. Preoperative transthoracic echocardiography confirmed a perimembranous VSD with a basal diameter of 8.5 mm and a left‐to‐right shunt width of 5.0 mm, along with a small patent foramen ovale (PFO, 2.8 mm) and mild tricuspid regurgitation. The child was asymptomatic, with a Ross score of 1, indicating preserved cardiac function.

## 3 Differential Diagnosis, Investigations and Treatment

Differential diagnoses included neuromuscular disorders causing ptosis, immune dysfunction related to prior hematopoietic stem cell transplantation, and congenital cardiac defects responsible for the murmur. Preoperative investigations consisted of echocardiography confirming a perimembranous ventricular septal defect, hematologic and immune assessments demonstrating complete remission and full immune reconstitution, and neuromuscular evaluation showing stable ocular myasthenia gravis without bulbar involvement.

On May 28, 2025, the patient underwent surgical repair of a perimembranous VSD under general anesthesia. Induction was achieved with amobarbital (0.1 mg/kg/h continuous infusion) and esketamine (0.5 mg/kg single dose), while cisatracurium (1.5 mg/kg) was used cautiously for muscle relaxation. Cardiopulmonary bypass was established via aortobicaval cannulation with a flow rate of 80–100 mL/kg and a systemic temperature of 32°C. HTK cardioplegia (50 mL/kg) was infused over 6 min for myocardial protection.

**FIGURE 1:**
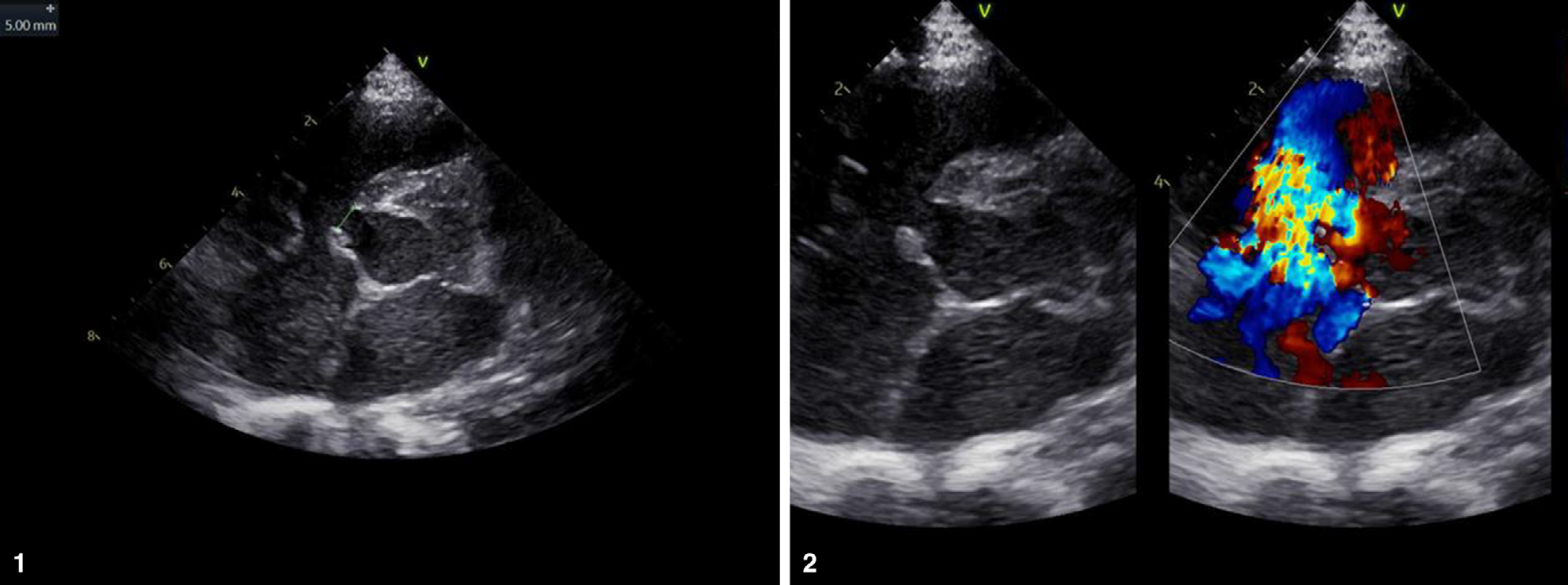
Preoperative transthoracic echocardiogram showing a perimembranous VSD with left‐to‐right shunt (arrow).

Intraoperative findings revealed a perimembranous VSD (8.5 mm basal diameter, 5.0 mm shunt width), a patent foramen ovale (2.8 mm), and mild tricuspid regurgitation (Figure 1). The VSD was repaired using a bovine pericardial patch, and the PFO was closed directly.

Thymectomy was performed concurrently, considering evidence that thymus removal may benefit MG and that it also facilitates surgical exposure during cardiac procedures. The thymus was not submitted for pathological examination.

Cardiopulmonary bypass time was 46 min, and aortic cross‐clamp time was 23 min. The patient was extubated 2 h postoperatively, maintaining stable spontaneous respiration without evidence of myasthenic crisis. Oral pyridostigmine was resumed at the preoperative dose. Cefuroxime was administered prophylactically for 48 h as standard empirical coverage.

## 4 Conclusion and Results (Outcome and Follow‐Up)

**FIGURE 2:**
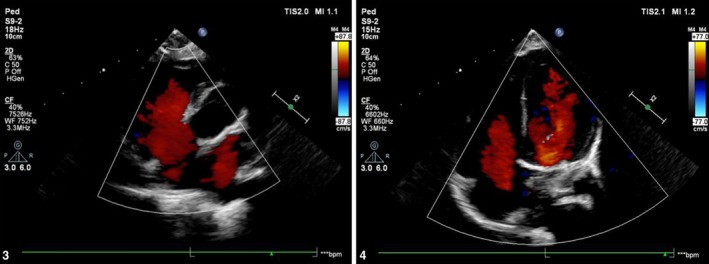
Postoperative echocardiogram confirming successful VSD closure and normal ventricular function.

The postoperative course was uneventful. The patient was discharged on postoperative day 7, and follow‐up echocardiography confirmed complete VSD closure with preserved biventricular function (Figure 2) (Table 1).

**TABLE 1:** Echocardiographic comparison before and after VSD repair.

Parameter	Preoperative findings	Postoperative findings
LVEF (%)	68%	66%
LA (mm)	26.7	22.9
AO (mm)	18.0	15.8
LVEDD (mm)	39.5	34.8
LVESD (mm)	24.9	22.6
Interventricular Septum	Perimembranous defect (8.5 mm basal, 5.0 mm shunt width), left‐to‐right flow with partial shunt into RA	Patch echo visible at perimembranous septum, no residual shunt detected
PFO	2.8 mm, left‐to‐right shunt	Intact atrial septum, no abnormal flow
Tricuspid Valve	Mild regurgitation	Mild regurgitation persists

This case report demonstrates the successful surgical repair of a ventricular septal defect in a young child with coexisting ocular myasthenia gravis and a history of hematopoietic stem cell transplantation for myeloid sarcoma, a combination of conditions not previously described in this context. The favorable outcome was contingent upon a meticulously orchestrated, multidisciplinary approach. Key elements contributing to success included: (1) comprehensive preoperative assessment and planning by a dedicated team encompassing cardiology, cardiac surgery, anesthesia, neurology, and hematology/oncology; (2) an anesthetic strategy rigorously minimizing and meticulously monitoring neuromuscular blocking agents, alongside avoidance of MG triggers; (3) tailored infection control protocols commensurate with the patient's post‐HSCT immune reconstitution status; and (4) vigilant perioperative monitoring with prompt management of potential complications.

This experience provides a valuable framework for managing pediatric patients with similarly complex, multi‐system comorbidities requiring major cardiac or non‐cardiac surgery, emphasizing that safety is achievable through individualized care and seamless interdisciplinary collaboration.

## 5 Discussion

This report details the successful surgical repair of a VSD in a 3 year‐old child presenting with the rare triad of congenital heart disease, ocular myasthenia gravis (OMG), and a history of hematopoietic stem cell transplantation (HSCT) for myeloid sarcoma. To our knowledge, this represents the first documented case of cardiac surgery in a pediatric patient with this specific combination of comorbidities. The favorable outcome underscores the paramount importance of meticulous, multidisciplinary perioperative management tailored to address the unique challenges posed by each condition and their potential interactions.

### 5.1 Managing Ocular Myasthenia Gravis in the Perioperative Setting

The primary concern with OMG centered on the heightened sensitivity to nondepolarizing neuromuscular blocking agents (NMBAs) and the risk of postoperative respiratory compromise or myasthenic crisis [4]. Our anesthetic strategy prioritized minimizing NMBA exposure. Cisatracurium was selected due to its intermediate duration, organ‐independent metabolism (Hoffman elimination), and potentially more predictable reversal profile in MG compared to other agents, albeit still requiring extreme caution [4]. Avoidance of known triggering agents (e.g., aminoglycosides, magnesium) was mandated. The continuation of pyridostigmine throughout the perioperative period and the absence of any bulbar/respiratory symptoms preoperatively were likely key factors in the rapid, uneventful extubation at 2 h and the absence of MG exacerbation. While ocular MG is generally considered less severe than generalized forms, this case reinforces that vigilance and specialized anesthetic planning are essential even in MGFA class I patients undergoing major surgery.

### 5.2 Navigating the Post‐HSCT Immunological Landscape

Despite documented complete immune reconstitution and the absence of GVHD or immunosuppression at the time of surgery, the inherently altered immune competence following HSCT necessitated aggressive infection prophylaxis and surveillance [5]. This included strict adherence to aseptic techniques, protective isolation measures in the PICU, and the use of prophylactic antibiotics (cefoperazone‐sulbactam for 48 h) chosen for broad Gram‐negative coverage relevant to our institutional epidemiology. Meticulous monitoring for signs of infection (fever, leukocyte shifts) and potential GVHD flare was maintained. The decision against prolonged or broader‐spectrum antimicrobials or antifungal prophylaxis was based on his robust immune parameters and the absence of specific risk factors (e.g., recent neutropenia, active GVHD). The uneventful recovery without infectious complications validates this risk‐adapted approach but highlights the critical need for individualized assessment of immune status in post‐HSCT surgical patients.

### 5.3 The Role of Thymectomy and Multidisciplinary Collaboration

Thymectomy was performed during the VSD repair. While thymectomy is a well‐established treatment for generalized MG, particularly in acetylcholine receptor antibody‐positive patients, its role in pediatric ocular MG, especially in very young children, remains controversial and less clearly defined [6, 7]. The decision in this case was influenced by several factors: (1) the theoretical potential for immunomodulation and long‐term benefit in MG, even in ocular forms; (2) the relative ease of access during median sternotomy for cardiac surgery, minimizing additional morbidity; and (3) the absence of contraindications. Long‐term follow‐up will be necessary to assess any impact on his OMG course. The successful navigation of this complex case hinged fundamentally on close collaboration between pediatric cardiothoracic surgery, cardiac anesthesia, pediatric neurology (MG expertise), hematology/oncology (transplant expertise), intensive care, and nursing. Preoperative MDT meetings established consensus on critical pathways: individualized anesthesia (NMBA strategy, avoidance lists), infection control protocols, pyridostigmine management, and contingency plans for MG crisis or infection. Seamless communication and shared goals persisted throughout the perioperative period.

### 5.4 Generalizability and Limitations

This single case report demonstrates the feasibility of safe cardiac surgery in children with exceptionally rare and complex comorbidities like concurrent OMG and post‐HSCT status. Key transferable principles include:

Mandatory Preoperative MDT Assessment: Involving all relevant specialties for risk stratification and joint planning.

Anesthesia Precision: Meticulous NMBA selection, dosing and avoidance of triggers in MG.

Stringent Infection Control: Tailored to the individual's post‐HSCT immune status.

Continuity of Chronic Disease Management:

Seamless continuation of medications like pyridostigmine.

Vigilant Postoperative Monitoring: Focused on organ systems at highest risk (respiratory in MG, infectious/immune in HSCT).

The primary limitation is inherent to case reports: the experience with a single patient. Larger studies are impractical given the rarity, making detailed reporting of successful management strategies valuable for similar future cases.

Furthermore, recent advances in the Medical Internet of Things (IoT) have revolutionized the landscape of oncology follow‐up and survivorship care. IoT‐based health systems integrate wearable sensors, wireless data transmission, and artificial intelligence analytics to continuously monitor physiological and immunologic parameters in cancer patients, enabling early detection of complications and facilitating timely interventions [8].

In pediatric oncology, particularly among long‐term survivors after hematopoietic stem cell transplantation (HSCT), IoT technology has demonstrated potential to enhance remote monitoring of immune recovery, detect infection or recurrence risk, and improve adherence to multidisciplinary evaluation [9]. These platforms can transmit real‐time clinical and laboratory data to healthcare providers, allowing proactive adjustments in therapy or prophylaxis.

Although IoT was not directly applied in the present case, integrating such smart surveillance systems into postoperative cardiac and oncologic follow‐up may further strengthen patient safety, optimize clinical outcomes, and establish a model for technology‐assisted precision care in children requiring both HSCT and cardiac surgery.

## Author Contributions

**Shuhan Rong:** data curation, formal analysis, investigation, writing – original draft. **Wanyu Xu:** investigation, methodology, resources. **Xin Li:** writing – review and editing.

## Funding

The authors have nothing to report.

## Ethics Statement

As a single‐case report with the patient's signed consent, no other ethical review was required.

## Consent

Written informed consent from the patient's legal guardian for publication, including images, is in accordance with institutional and journal ethics policy.

## Conflicts of Interest

The authors declare no conflicts of interest.

## Data Availability

The data that support the findings of this study are available from the corresponding author upon reasonable request.
